# Minimization of Radiation Exposure due to Computed Tomography in Inflammatory Bowel Disease

**DOI:** 10.5402/2012/790279

**Published:** 2012-04-10

**Authors:** Patrick D. Mc Laughlin, Owen J. O'Connor, Siobhán B. O'Neill, Fergus Shanahan, Michael M. Maher

**Affiliations:** ^1^Department of Radiology, University College Cork, Cork, Ireland; ^2^Department of Medicine, University College Cork, Cork, Ireland; ^3^Alimentary Pharmabiotic Centre, University College Cork, Cork, Ireland

## Abstract

Patient awareness and concern regarding the potential health risks from ionizing radiation have peaked recently (Coakley et al., 2011) following widespread press and media coverage of the projected cancer risks from the increasing use of computed tomography (CT) (Berrington et al., 2007). The typical young and educated patient with inflammatory bowel disease (IBD) may in particular be conscious of his/her exposure to ionising radiation as a result of diagnostic imaging. Cumulative effective doses (CEDs) in patients with IBD have been reported as being high and are rising, primarily due to the more widespread and repeated use of CT (Desmond et al., 2008). Radiologists, technologists, and referring physicians have a responsibility to firstly counsel their patients accurately regarding the actual risks of ionizing radiation exposure; secondly to limit the use of those imaging modalities which involve ionising radiation to clinical situations where they are likely to change management; thirdly to ensure that a diagnostic quality imaging examination is acquired with lowest possible radiation exposure. In this paper, we synopsize available evidence related to radiation exposure and risk and we report advances in low-dose CT technology and examine the role for alternative imaging modalities such as ultrasonography or magnetic resonance imaging which avoid radiation exposure.

## 1. Introduction

Increased exposure to ionising radiation in patients with Crohn's disease has been documented in recent publications and is a significant cause for concern [1, 2]. Improvements in CT hardware and software have greatly expanded its role in the diagnosis and characterisation of IBD, the detection of complications, and the assessment of response to treatment [2]. These advances have been hugely beneficial to the management of many patients, but CT may on occasion become a victim of its own success when IBD patients may undergo CT examination for a less than robust indication and the radiologist's report may not have any impact upon patient management. In these cases, the potential for carcinogenesis as a result of radiation exposure is difficult to justify.

## 2. Ionizing Radiation: Potential Hazards in IBD Patient

Cancer induction is the primary concern for IBD patients who are routinely exposed to ionizing radiation. IBD patients can be subjected to serial imaging studies over prolonged periods of followup due to early age of presentation and, sometimes, decades of active disease [2]. In addition, IBD patients are already at increased risk of certain malignancies such as colorectal carcinoma and small bowel adenocarcinoma and these risks could potentially be compounded in patients with more severe disease and by use of immunomodulatory drugs.

Carcinogenesis is a stochastic effect of X-radiation; that is to say it is random. Cancer induction does not exhibit an upper or lower threshold of occurrence, and the probability of cancer induction is variable [3]. It is noteworthy that there is no X-radiation dose where cancer induction may not occur. Greater exposure to CT, however, is believed by many to increase the likelihood of carcinogenesis in IBD patients, but carcinogenesis typically occurs many years remote from the exposure [4]. Similarly, exposure to ionizing radiation in early life is believed to magnify the risk of tumour induction [5]. It has been reported that patients who are diagnosed with Crohn's disease as children are more likely to receive higher lifetime cumulative radiation exposures [2, 5, 6]. Children undergoing CT receive greater organ doses than adults, and, in children, most organs are more sensitive to radiation-induced cancer than in adults [5]. Thus, particular care needs to be taken in imaging of all Crohn's disease patients, particularly in those where the initial diagnosis was made as a child.

## 3. Computed Tomography and Patient Dose

When an X-ray beam interacts within a patient, energy is deposited in irradiated organs. The absorbed dose is a measure of the amount of energy deposited per unit mass. The effective dose (ED), measured in Sieverts (Sv), is a more useful unit that estimates the biologic detriment of a given absorbed dose. ED takes into account each organ exposed to ionizing radiation as well as each organ's radiosensitivity [7]. CT exposures by their nature are not uniformly distributed across the entire patient's body but are limited to the region of the patient's body that is being examined, for example, a patient's head during a CT brain. One of the primary benefits of ED as a unit is that it accounts for nonuniform doses, such as to a patient's head during CT brain, which are subsequently expressed as equivalent whole-body dose that would have the same risk of biologic effect. ED, therefore, allows more accurate comparison of the risk of biological effect between, for instance, a CT scan of brain and a CT scan of abdomen and pelvis [8]. ED, as a unit, also allows us to compare the risk of biologic effect between ionizing radiation doses delivered by different imaging modalities such as CT and nuclear medicine, plain radiography, and fluoroscopy.

A standard CT of abdomen and pelvis (CTAP) exposes the patient to an ED of approximately 8 mSv although values reported in literature range widely from 3.5 mSv to 25 mSv [9]. The radiation exposure associated with CTAP is substantial (8 mSv) when compared to chest radiograph (0.02 mSv), plain radiograph of the abdomen (PFA) (0.7 mSv), and barium studies (3.5–5 mSv) [9, 10].

## 4. Cumulative Radiation Exposure in Inflammatory Bowel Disease

A 15-year retrospective examination of abdominal imaging in a tertiary referral centre for Crohn's disease reported that subgroups of Crohn's disease patients were “at risk” for increased exposures to ionizing radiation [2]. The subgroups who were at greater risk of higher cumulative radiation exposure included patients in whom the diagnosis of Crohn's disease was made before 17 years of age, those with more severe disease requiring treatment with steroids or infliximab, and those requiring surgical intervention [2]. The average cumulative effective dosages per patient increased over the 15-year study period from 7.9 mSv in the initial 5-year period to 25 mSv in the final 5-year period [2]. This escalation was mainly attributed to increased use of CT, without a corresponding drop in the use of plain radiographs of the abdomen. Of the imaging studies performed during this period, 19.7% were CT scans, accounting for 84.7% of the diagnostic radiation exposure [2]. A cumulative effective dose of greater than 75 mSv, the effective dose equivalent of 3,750 standard chest X-rays, was noted in almost 16% of patients [2, 10]. Cumulative exposure to low-level ionising radiation of this magnitude has previously been estimated to increase mortality due to cancer by 7.3% [4].

## 5. Biologic Effects of Exposure to Ionizing Radiation

Exposure to ionizing radiation results in DNA damage which is intimately related to the appearance of gene or chromosomal mutations and therefore related to multistage cancer development. The pathway of cancer induction secondary to ionizing radiation exposure does not appear to differ from that which applies to spontaneous cancer or to cancers associated with exposure to other carcinogens [11].

Acute high-level exposure to ionizing radiation produces clearly meusurable consequences in humans, including cancer induction. In general, protracted exposures to the same total dose of X-radiation are associated with lower risks than those of an acute exposure, both for cancer and other endpoints [12]. Latency of cancer induction due to ionizing radiation exposure is well recognized, and available evidence indicates that the time from exposure to incidence of cancer for solid tumors is at least 5 years and more commonly 10–20 years [13].

The Life Span Study of a cohort of atomic bomb survivors is the epidemiological study with the highest statistical power for evaluating the effect and risks of low-dose ionizing radiation exposure [14]. Atomic bomb survivors with a dose range from 5 to 100 mSv (mean dose, 29 mSv) show a significantly increased incidence of solid cancer (*P* = 0.05) compared with the population who were exposed to <5 mSv [15]. 

It has been estimated that 29 000 future cancers could be related to CT scans performed in the US in 2007 [16] and this risk may be three to five times higher for children [17].

## 6. What Can We Do?

The best way of protecting patients from the potentially detrimental effects of ionising radiation is to limit their exposure to ionising radiation in every available way. CT has been reported to represent 10% of all studies using ionising radiation, while accounting for two-thirds of the overall radiation dose to patients [18]. The three principal ways of reducing the overall radiation dose from CT in the population include limiting the use of CT scanning to those clinical scenarios where the examination is unequivocally indicated and likely to change patient management, the use, where feasible, of alternative imaging modalities such as ultrasonography and MRI which do not result in exposure to ionising radiation, and when CT is indicated, the use of low-dose CT protocols, which utilise all available CT technology developed for radiation dose optimisation, ensuring that a diagnostic quality CT study is acquired at lowest possible radiation exposure [19].

## 7. Low-Dose CT Technology

An emerging method of limiting radiation exposure in populations requiring frequent imaging is development of disease-specific low-dose CT protocols [20]. Low-dose CT protocols have been successfully developed which preserve diagnostic yield at significantly reduced radiation exposure, and this has been particularly effective in CT scanning of the thorax [21]. Achieving diagnostic quality low-dose CT in patients with IBD is particularly challenging as abdominal and pelvic imaging does not lend itself as well to low radiation dose scanning as the thorax. CT in the abdomen and pelvis requires good image contrast to resolve the pathological changes which occur in the liver, spleen, and kidneys. The slight increases and decreases in attenuation value that represent pathology can be obscured by increased image noise more so than in the thorax, where tissues have greater inherent contrast due to the large difference in their densities [22].

In the past, fixed tube kilovoltage and amperage settings were used in CT of the abdomen and pelvis; this resulted in areas such as the mid-abdomen receiving the same exposure as regions such as the pelvis. This was an inefficient method of acquisition, and some regions were overirradiated, without any benefit in terms of image quality while other regions were potentially underexposed, increasing image noise and reducing image quality [23].

Automatic tube current modulation (ATCM) in CT was a major development of the last decade [20]. This technology adjusts tube current during a CT scan depending on X-ray attenuation in that anatomic location, and tailors the dose output of the CT tube to patient size and shape. This method ensures that thicker regions of the body are imaged using higher tube currents than thinner, less attenuating areas. Within each imaged section, user-specified noise indices are chosen, which predict an acceptable level of image noise, thereby preserving diagnostic quality while minimising radiation exposure [24, 25]. The higher the noise index chosen by the radiologist, the greater the noise in the CT image and the lower the radiation dose required to acquire the image. Studies which investigated the effectiveness of ATCM for optimising radiation exposure in CT have reported that a reduction in dose can be achieved in 87% of examinations using ATCM, with an average tube-current time product reduction of 32% [24].

Reductions in CT dose often have a negative impact on the diagnostic quality of the images due to increasing noise. Noise is a statistical variation in the attenuation values not reflecting the underlying anatomy which can result in a lack of clear contrast between two adjacent tissues and blurring of the anatomic features of the image. As previously stated, noise can be particularly problematic in the solid upper abdominal organs such as the liver, spleen, and kidneys [22]. In patients with IBD where CT findings are most commonly limited to the small and large bowel, a larger amount of image noise has been found to be diagnostically acceptable. In a single series, elevating the noise index to 18–25 yielded a 31–64% reduction in radiation dose while preserving diagnostic accuracy [26].

At present many noise reduction strategies are being developed, with varied success, to maintain image quality while significantly reducing radiation dose. The earliest strategies included the use of noise reduction filter (NRF) software applications which are used to postprocess CT images acquired at significantly reduced radiation dose, improving their diagnostic quality by reducing image noise [22, 27]. NRFs were shown to be effective at reducing image noise, but there is potential for negative impact on diagnostic quality of images by reduced lesion conspicuity in organs such as the liver by “over-smoothening” [27].

More promising noise and, therefore, dose reduction strategies involve improvements in the image reconstruction process at the time of CT acquisition. The choice of image reconstruction algorithm is critical to the quality and appearance of CT images [28]. 

Although iterative image reconstruction algorithms were used to generate images in the first commercial clinical CT scanner [29], filtered back projection (FBP) became ubiquitous as it is a more rapid and more computationally efficient method with relatively low mathematical demands [30]. However, FBP is not well suited to low-dose CT where data within the image is limited and noise is high [31]; this has led more recently to renewed research and commercial interest in refining methods of iterative reconstruction for reducing noise in images acquired at lower radiation dose. Methods of iterative reconstruction currently represent the most exciting dose optimizing developments in CT [32]. Various modifications of iterative reconstruction are being developed and refined by different CT manufacturers including Adaptive Statistical Iterative Reconstruction (ASIR) (General Electric Healthcare, Milwaukee, WI), Iterative Reconstruction in Image Space (IRIS) (Siemens Healthcare, Erlangen, Germany), Adaptive Iterative Dose Reduction (AIDR) (Toshiba Medical Systems, Tustin, CA), and iDose (Phillips Healthcare, Best, The Netherlands).

Adaptive statistical iterative reconstruction (ASIR) is a noise-efficient reconstruction algorithm [33, 34] which is computationally fast and is proven to result in images with good low-contrast detail, preserved image quality, and with typical radiation dose reductions of greater than 30% [35]. Pilot studies with ASIR found that radiation dose can successfully be reduced by 50% in CT colonography [36], 44% in coronary CT angiography [37], and approximately 50% in CT abdomen and pelvis [38] without significantly affecting image quality.

The next step in optimising image quality in studies acquired at significantly reduced radiation dose is the ongoing development of advanced generations of iterative reconstruction such as Model-Based Iterative Reconstruction (MBIR), which is being developed by GE Healthcare. MBIR can incorporate a physical model of the CT system into the reconstruction process to characterize the data acquisition process, including noise, beam hardening, and scatter [39]. MBIR offers the potential to further enhance image quality at even lower radiation doses than ASIR. However, due to limitations in computing power and reconstruction technology, model-based iterative approaches have not been practical for commercial CT scanners until recently, though studies refining this technology with the aim of introducing it into clinical practice are now in progress.

Despite emerging advanced reconstruction and processing techniques, it is imperative that dose reduction should also be achieved by optimizing basic acquisition parameters during the day to day practice of CT. Human errors in CT planning have recently been publically highlighted resulting in gross dose increases [40]. These errors emphasize the importance of the role of the CT technologist in the careful planning and design of CT protocols that prioritize dose optimisation in day to day practice. As an example, scanning beyond the anatomical limits of the imaging examination in the context of CT of abdomen and pelvis has been demonstrated to be common practice, with 97% of cases having extra images above the diaphragm and 94% having extra images below the symphysis pubis in a published study [41]. In the above study, the additional images added little in terms of extra diagnostic information but added significantly to the radiation dose imparted. This study therefore emphasises that the goal of radiation dose optimisation requires a multidisciplinary approach and radiologists, CT technologists, medical physicists, and referring physicians must be alerted to the fact that attention to detail, and fine adjustments in practise can have a major impact in this area.

## 8. Alternatives to CT

In an effort to reduce cumulative radiation exposure to patients with IBD, many centres currently utilise imaging techniques such as magnetic resonance imaging (MRI) and ultrasound, which do not result in exposure to ionising radiation, to complement or replace CT and barium studies. In IBD patients, MR enterography offers excellent image quality, equal to or often better than barium or CT studies, and, in most clinical settings, represents a realistic alternative to these studies for examination of the small and large bowel [42]. Evaluation of small bowel disease has traditionally been carried out by barium follow-through, which has an associated effective radiation exposure of 3 mSv [10]. When MR studies with oral contrast, performed primarily for small bowel evaluation, were compared retrospectively with colonoscopy as standard for large bowel disease identification, sensitivity of 80% and specificity of 100% for the detection of colitis were demonstrated, with a positive predictive value of 1.0 and a negative predictive value of 0.8 [42]. There has been some suggestion that the mucosal detail seen on MR is inferior to that seen on small bowel follow-through [43] but MR imaging performed after the administration of intravenous contrast agents permits the identification of abnormal mucosal enhancement patterns which are key indicators of active inflammation. Prospective comparison of CT and MR enterography and small bowel follow-through, with optical ileocolonoscopy as reference standard, showed each modality was equally accurate at detecting active Crohn's disease of the small bowel, but the sensitivity values for CT and MR enterography for the detection of extraenteric complications were significantly higher than those for small bowel follow-through [43]. Therefore, gastrointestinal imaging with MR has the potential to safely reduce radiation exposure in patients with IBD with comparable or even superior diagnostic effectiveness to CT. MRI may prove particularly useful for follow-up imaging, for the assessment of treatment response, for imaging of the terminal ileum and caecum following incomplete colonoscopy, and for imaging of nonacute patients. Residual intraluminal air, long examination times, increased cost, and reduced out-of-hours access represent obstacles to the replacement of CT by MRI in sick patients with acute disease exacerbations [44]. In the critically ill patient, for a number of reasons including examination duration and difficulty in monitoring ill patients in MRI scanners, MRI, compared with CT, is much more difficult to perform.

The intestinal and extraintestinal manifestations of IBD can also be evaluated using ultrasound, again avoiding exposure to ionising radiation [44]. Ultrasound is used to image the small and large bowel with sensitivities of 78–96%, and 86–100%, respectively, for bowel pathology [44, 45]. While useful in assessment for strictures and fistulae, and extraintestinal manifestations such as gallstones and renal calculi, ultrasound appears best able to examine the terminal ileum and caecum [44]. However, most experts acknowledge that the use of ultrasound in imaging of IBD patients is increasingly limited by patient body habitus and, being inherently operator dependent [44, 45], the feasibility of using ultrasound to examine the bowel wall for disease activity varies significantly from centre to centre. The use of contrast agents with ultrasonography for examination of the bowel could also be indicated in the followup of patients with Crohn's disease as this practice has demonstrated comparable sensitivity to small bowel enema for the detection of small bowel lesions, as well as strictures and prestenotic dilatation [46]. Most radiologists specialising in gastrointestinal imaging would acknowledge, however, that ultrasound is unlikely to fully replace barium examinations or CT in imaging of IBD patients, unlike MR enterography which could potentially achieve this aim if elimination of radiation exposure associated with diagnostic imaging was identified as a major priority and the necessary investment in MR imaging was made.

## 9. Education in Radiation Exposure Associated with Diagnostic Imaging

When assessed with regard to their knowledge of radiation exposure and the associated risks, clinicians consistently perform disappointingly with significant underestimation of typical radiation exposures associated with different diagnostic imaging studies [47–50]. The European Council issued the Euratom directive in 1997 and advised integration of a module in radiation protection, into the curriculum of medical schools in an effort to raise awareness [51]. Recent evaluation of medical students, undertaken following curriculum change which included modules in diagnostic imaging, reassuringly demonstrates an incremental expansion in students' knowledge of radiation protection with each year of medical training [52]. In the United States, the Image Gently campaign was established to increase public awareness of radiation exposure in children [53, 54]. Along with this, Strauss et al. suggest ten steps to optimise image quality and lower CT dose in paediatric patients [55], principles which can largely be applied to imaging of all patients. A proposed means of raising awareness of ionising radiation exposure among patients with IBD and their clinicians, as well as allowing monitoring of cumulative dose, is the development of a radiation diary [44]. Experts in the field of radiation dose optimisation have advocated colour-coding of imaging studies in an effort to highlight those studies that are associated with exposure to ionising radiation (red) and those that are not (green) [56].

Selection of the appropriate imaging modality for the appropriate indication is fundamental. In recent years, patient care is increasingly adopting a multidisciplinary approach, therefore collaboration between clinicians and radiologists is required for creation of agreed imaging protocols, and patient management and investigation planning should include consideration of prior cumulative radiation exposure. Close interaction may help better differentiate the clinical indications which justify ionising radiation exposure with CT from those situations where the use of modalities such as MR or ultrasound may be more appropriate.

## 10. Conclusion

There is a broad range of imaging modalities available for the investigation and management of IBD. Use of CT has expanded in recent years, with increased availability and advances in CT hardware and software technology significantly improving the diagnosis of suspected IBD, the evaluation of complications, and the response to therapy. However, concern is mounting worldwide regarding increasing usage of CT and the rapidly growing contribution of CT scanning to radiation exposure of the entire population [19]. This issue is particularly important to a number of patient groups including IBD patients who are “at risk” for significant cumulative radiation exposure from diagnostic imaging due to the chronicity of their disease. This radiation exposure may compound risk for future cancers in this already predisposed patient population. There is increasing awareness of the importance of rationalisation of radiation exposure in IBD patients, to provide the best quality diagnostic images for the lowest possible dose to the patient. The optimal strategy for minimization of radiation exposure in IBD has not yet been determined but requires a multifaceted collaborative approach. Modification of referral practices among clinicians, impacted upon by educational initiatives which highlight the causes and consequences of diagnostic imaging-related ionizing radiation exposure, has a large part to play in limiting the number of patients unnecessarily exposed to a significant radiation burden. Radiologists, in turn, need to integrate low-dose CT imaging protocols, which utilise latest technological developments, into routine practice, particularly in those patients requiring serial imaging studies, minimising the CT-related dose to individual patients with each scan performed. The use of alternative imaging modalities that do not result in radiation exposure, such as ultrasound and MR, should be encouraged whenever possible.

##  Conflict of Interests

No potential conflict of interests relevant to this paper was reported.
